# CO_2_ Laser Photoacoustic Spectrometer for Measuring Acetone in the Breath of Lung Cancer Patients

**DOI:** 10.3390/bios10060055

**Published:** 2020-05-27

**Authors:** Mitrayana, Donni Kis Apriyanto, Mirza Satriawan

**Affiliations:** 1 Department of Physics, Universitas Gadjah Mada, Yogyakarta 55281, Indonesia; mirza@ugm.ac.id; 2 Department of Physics, University of Lampung, Bandar Lampung 35141, Indonesia; donni.kis@fmipa.unila.ac.id

**Keywords:** lung cancer, acetone, volatile organic compounds, CO_2_ laser photoacoustic

## Abstract

A CO_2_ laser has the advantages of being high in power and having many laser lines in the 9–11 µm infrared region. Thus, a CO_2_ laser photoacoustic spectrometer (PAS) can have a multi-component measurement capability for many gas compounds that have non-zero absorption coefficients at the laser lines, and therefore can be applied for measuring several volatile organic compounds (VOCs) in the human breath. We have developed a CO_2_ laser PAS system for detecting acetone in the human breath. Although acetone has small absorption coefficients at the CO_2_ laser lines, our PAS system was able to obtain strong photoacoustic (PA) signals at several CO_2_ laser lines, with the strongest one being at the 10P20 line. Since at the 10P20 line, ethylene and ammonia also have significant absorption coefficients, these two gases have to be included in a multi-component measurement with acetone. We obtained the lowest detection limit of our system for the ethylene, acetone, and ammonia are 6 ppbv, 11 ppbv, and 31 ppbv, respectively. We applied our PAS system to measure these three VOCs in the breath of three groups of subjects, i.e., patients with lung cancer disease, patients with other lung diseases, and healthy volunteers.

## 1. Introduction

In the last several decades, the gas chromatography - mass spectrometry (GC-MS) method has become the common method used for the study of the volatile organic compound (VOC) in the human breath [1,2]. Unfortunately, the GC-MS method is considered not practical, needs thorough sample preparation and experts for operation [3]. It method is also unreliable for detecting gas concentration less than ppbv. Moreover, the detection of these VOC gases needs to be performed in a one-time setup for several gases, which is difficult to be realized in the GC-MS method. These reasons motivate many scientists to develop more practical and highly sensitive tools for detecting VOCs in human breath. Some of those tools are enhancement and perfection of the mass spectroscopy methods, like the Selected Ion Flow Tube (SIFT)-MS [4,5], and the Proton Transfer Reaction (PTR)—MS [6]. Other use different approach, like the laser spectroscopy method, that uses the electromagnetic radiation absorption of the targeted gas compound. The sensitivity of the detection in the laser spectroscopy method can be increased using some methods, like Multi-pass Cell Spectroscopy [3], Cavity Ring-Down Spectroscopy (CRDS) [7], and Photo Acoustic Spectroscopy [8].

Photoacoustic spectroscopy (PAS) method is considered to be reliable in detecting trace gases directly with simple sample preparation [9]. PAS is based on the concept of generating acoustic pressure waves from certain targeted gas molecules [9]. The molecules absorbed electromagnetic radiation and released the energy in the form of collisions to other surrounding molecules, and this will then heat the surrounding gas. Modulating the electromagnetic radiation at acoustic frequency will modulate the heat at the same frequency and thus creating acoustic pressure waves, which can be detected using microphones. In order to increase the sensitivity of the microphone detection, the whole process of acoustic pressure waves generation should take place inside an acoustic resonance cell, i.e., the photoacoustic cell. If the modulated acoustic waves have the same frequency as the resonance frequency of the PA cell, the acoustic signal detected by the microphone will increase. The targeted gas may have large absorption coefficients at certain characteristic wavelengths, thus by using radiation source whose wavelength is match with the characteristic wavelength of the targeted gas, one can selectively detect the targeted gas compound. For the infrared spectrum region, these characteristic wavelengths correspond to the vibrational frequencies of the gas compound. The produced PA signal is proportional to the targeted gas concentration, the gas absorption coefficient, and the power of the radiation source. Even though usually one uses the characteristic wavelength of the targeted gas, it is actually possible to use other wavelength to produce the PA signal, as long as the gas has non-zero absorption coefficient at that wavelength. But in this case, the selectivity capability is loss.

In the last two decades, there have been many studies on the PAS method with various radiation sources for detecting several VOCs in human breath. Among the radiation sources for the PAS system, the CO_2_ laser has many advantages of being high in power with many easily tunable laser lines in the infrared region. There are several reports on the measurement of VOCs in the human breath using a CO_2_ laser PAS system, especially for measuring ethylene and ammonia [10,11,12,13,14,15,16,17]. Those are because the ethylene and ammonia have strong absorption coefficients at several CO_2_ laser lines. Ammonia is one of the major compound in the human breath, with a typical concentration in human breath around 422–2389 ppbv [18]. An increased concentration of ammonia in the human breath is thought to be related to several diseases like a renal failure [19], liver dysfunction [20], and Alzheimer’s disease. Dumitras, et. al. have measured the absorption coefficient of ammonia at several CO_2_ laser lines, with the largest is α = 57.12 cm^−1^ atm^−1^ at 9R30 line [12]. Unlike ammonia, ethylene occurs in smaller amounts in the human breath, but its concentration in a healthy human can reach several tens ppbv [21]. The trace of ethylene has been measured using the CO_2_ laser PAS system for many applications because one of the strongest absorption coefficients of ethylene coincides with the CO_2_ laser line, i.e., the 10P14 where α = 30.4 cm^−1^ atm^−1^. An increase in ethylene breath concentration is linked to oxidative stress in a person, like in patients on hemodialysis [16], inflammatory disorder [22], and ultraviolet damage of the skin [23]. This ethylene production has been attributed to the lipid peroxidation [22].

A multicomponent detection of breath VOCs using the CO_2_ laser PAS system was first done by Popa et al., who have measured ammonia and ethylene from patient breath with renal failure [15]. Recently, Popa et al. have used a CO_2_ laser PAS system for multi-component detection of carbon dioxide, ammonia, ethanol, methanol, and ethylene in the mouth breathing vs nasal breathing study [24]. Ammonia, ethanol, methanol, and ethylene are among major VOCs in human breath with a typical concentration of up to 100 ppbv. Besides these four gases, other major breath VOCs with typical concentration up to 100 ppbv are methane, hydrogen sulfide, carbon monoxide, acetone, and isoprene. Base on IR spectrum data from NIST [25], among these other VOCs, acetone has significant, although small absorption coefficient at the CO_2_ laser lines. Acetone has a characteristic absorption coefficient α = 0.27 cm^−1^ atm^−1^ (calculated from acetone infrared absorbance spectrum in [25]) at the wavelength of 9.166 µm, corresponds to a weak 9R42 line of the CO_2_ laser. In the 10–11 µm region, acetone has a small but non-zero absorption coefficient around α = 0.1 cm^−1^ atm^−1^ [25]. Together with its large typical concentration in the breath, acetone should be detectable using the CO_2_ laser PAS system.

There have been many studies regarding acetone concentration in the human breath. Turner, et al., using the SIFT-MS method, measured the breath acetone concentrations in the healthy human, the breath acetone concentration in healthy person falls in the range 148 to 2744 ppbv [26]. Schwarz et al. have studied the variations of breath acetone concentrations with age, gender and body-mass index (acetone concentration range: 281 ppbv to 1246 ppbv for subjects with no dietary control) [27]. Spanel et al. have shown that breath acetone has a wide variation of concentration due to diurnal increase and varying diet [28]. With this wide variation of concentration in human breath, it is debatable whether acetone can be used as a biomarker of some disease.

Nevertheless, breath acetone has been considered as a potential biomarker of some diseases. For example, it is known that there is a significant increase of acetone concentration in the breath of a patient with diabetes [29,30], There have been many studies using many methods for detecting acetone in human breath. Among those many methods, only Tyas et al. that has reported the use of CO_2_ laser PAS for measuring breath acetone concentration, where they measured the acetone concentration in the breath of a patient with type II diabetes mellitus, and obtained increased acetone concentration on the breath of type II diabetes mellitus patients with acetone concentration in the range of 1.01–1.62 ppmv compared to 0.15–0.85 ppmv in the healthy group [31]. Another interesting case is the acetone concentration in the breath of a patient with a lung cancer disease. There are various conflicting reports in the literature about the concentration of acetone in the breath of lung cancer patients. Bajtarevic et al. reported that the acetone concentration in the breath of lung cancer patients is somewhat less than in healthy patients [32]. Oppositely, Ulanowska et al. reported that the acetone concentration is relatively high in the breath of lung cancer patients compared to healthy patients [33]. Kischkel et al., on the other hand, reported a significantly larger acetone concentration in the breath of lung cancer patients compared to the smokers, but not significantly larger compared to the healthy patient [34].

In this paper, we present our study of using CO_2_ laser PAS system to detect acetone, and its application for detecting breath acetone in the lung cancer patients. Even though acetone has some large absorption coefficient at the 9R42 CO_2_ lines, we did not use this line for detecting acetone, since at that line the ethanol and methanol have strong absorption coefficients also (α = 2.0 cm^−1^ atm^−1^ and 0.1 cm^−1^ atm^−1^, respectively [25]), moreover, the 9R42 CO_2_ laser line had relatively low power. Instead, we used the strongest line of the CO2 laser, i.e., the 10P20 lines for detecting acetone. Since at 10P20 ammonia and ethylene also had strong absorption coefficients (α = 0.2 cm^−1^ atm^−1^ and 1.84 cm^−1^ atm^−1^, respectively [12,35]), we included these two gases in a multicomponent measurement together with the acetone. We chose the 10R14 and 10P14, where the ammonia and ethylene had strong absorption coefficients [12,35], while other major breath compounds had relatively small ones [25]. In the measurement of a sample that has many compounds like in the breath sample, one has to include all compounds that have non zero absorption coefficients at the laser lines that being used. The PA signal recorded at a line comes from the PA signal contribution from several gas compounds. For *N* gas compounds with a non zero absorption coefficient, one needs at least *N* laser lines where the *N* gas should have different absorption coefficients on those laser lines.

This study aims to show the possibility of detecting acetone, using the CO_2_ laser PAS system, together with ethylene and ammonia in a multi-component measurement setup, with its real-life application in the detection of breath acetone from lung cancer patients. Ethylene and ammonia in the breath are not directly related to lung cancer disease. Their inclusion in the measurement was required by the multicomponent PAS method, as described above.

As a comparison we also took breath samples from patients who have other lung diseases and from healthy patients (confirmed by their medical record). We only used a limited number of patients for this study: eleven patients who have lung cancer disease, nine patients who have other lung diseases, and ten healthy volunteers. The lung cancer patients were selected from the lung cancer patients of the Sardjito Hospital that did not have other lung disease. They did not have diabetes, renal disease, or inflammatory disorder. The patients who had other lung disease had bronchitis, pneumonia, or asthma, with no known additional chronic diseases. All non-healthy subjects were patients of Dr. Sardjito Hospital in Yogyakarta, Indonesia, and the ethical committee of Dr. Sardjito Hospital approved this study. Due to a limited number of patients and volunteers involved, this study was not aimed to show acetone as a potential biomarker for lung cancer.

## 2. Materials and Methods

**Figure 1:**
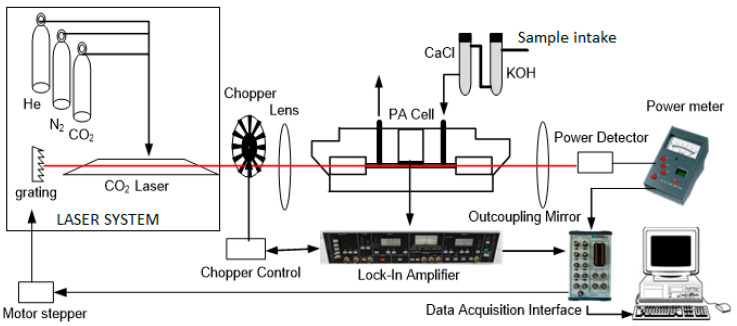
Schematic of the CO_2_ laser photoacoustic spectrometer.

The schematic of our lab-built CO_2_ laser PAS system is shown in Figure 1. The three main components of the system include the CO_2_ laser system, the photoacoustic (PA) cell system, and the electronics system (lock-in amplifier and the data acquisition interface).

Our CO_2_ laser was an axial flowing type gas laser, operating on a continuous wave mode at a tunable frequency using a grating, emitting radiation from many CO_2_ lines in the 9–11 μm regions. The CO_2_ laser used He, N_2_, and CO_2_ gases as active laser components, that were kept at a pressure of 30 mbar, 50 mbar, and 50 mbar, respectively. A power supply (HCN 350-20.000) was used to create an electrical discharge to excite the active laser gases. To optimize the laser power, there were several factors to be considered, namely setting the laser tube position alignment with the PA cell, controlling the composition ratio of the active ingredient of the CO_2_ laser, i.e., He, N_2_, and CO_2_, and the voltage and current regulation of the CO_2_ laser operation. For the laser operation, the current is set at 14.79 mA and the voltage at 9.61 kV with a negative polarity.

We used a PA cell with an intra-cavity setup where the cell was put inside the laser resonator. In this way, the laser light passed the PA cell several times, increasing the chance of laser light absorption by the gases inside the cell. The PA cell geometry was an H-type cylinder with a buffer at both ends that had windows positioned at a Brewster angle. The cylinder length was 100 mm, with its diameter was 6 mm. The buffer length was 50 mm, and its diameter 20 mm. Three microphones (Knowles EK 3033) were positioned in the middle of the cylinder symmetrically flushed on the cylinder wall. A chopper was placed in front of the CO_2_ laser tube to modulate the laser radiation. The chopping frequency of the chopper should be set to match the acoustic resonance frequency of the PA cell. The three microphones in the PA cell were connected to the lock-in amplifier (EQ & G model 5210) to amplify the signal with the same frequency of the chopper. A Zn Se lens was positioned in between the chopper and the PA cell, for focusing the laser beam into the PA cell. At the far end of the PA cell, after an outcoupling mirror, we positioned a power meter (OPHIR model AN2), for measuring the laser power.

Before applying the PAS system for measuring the VOCs concentration in the human breath, we conducted the calibration and characterization of the PAS system. These included: Scanning the CO_2_ laser lines to find the suitable line for acetone, ethylene, and ammonia; plotting the resonance curve to find the resonance frequency and the quality factor, i.e., the *Q* value of the PA cell; measuring the noise and the background signal; plotting the linearity curves of the PA signal versus the acetone, ethylene, and ammonia concentrations; and determining the lowest detection limit. As a reference standard, the various concentration of acetone, ethylene, and ammonia gases were obtained from the standard dilution process. The ethylene gas was provided by a certified local gas supplier, supplying a standardized concentration with a 99.95% purity. The acetone and ammonia gases were obtained from the standard solutions being vaporized. We performed a standard dilution procedure for those three gases to obtain different concentrations of the gases.

The volunteer’s breath was taken by asking them to exhale using their mouth into a container bag (Tedlar bag). Prior to the breath sample taking, the patients were required to not be taking any medication or drug on that day and to not eat anything for at least one hour before the samples were taken. All samples were then taken to the lab where measurements of acetone, ethylene, and ammonia concentrations were taken using the PAS system. Each breath sample was passed through the KOH and CaCl_2_ scrubber to remove the CO_2_ and the water vapor from the sample. The breath gas was then flown into the PA cell for the PA signal measurement after passing through the scrubber.

## 3. Results and Discussion

**Figure 2:**
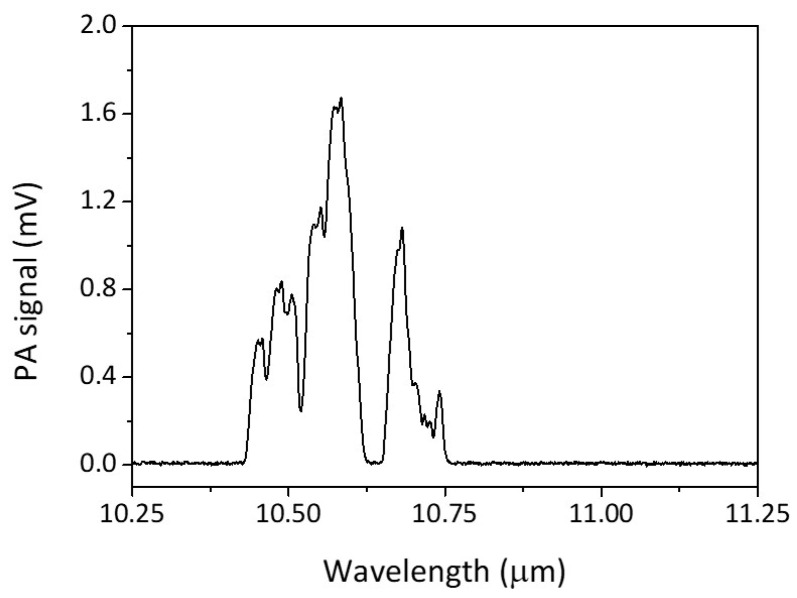
The scanning result of the photoacoustic (PA) signal of the acetone.

The scanning result of the CO_2_ laser wavelength with the acetone gases flowing inside the PA cell tube is shown in Figure 2. From Figure 2, it seems that there were several large PA signals at almost all the 10 μm region of the CO_2_ laser lines. The largest PA signal in Figure 2 corresponds to the CO_2_ laser line 10P20, i.e., at the wavelength of 10.59 μm.

The PA signal would be strong if the generated acoustic signal matched the resonance frequency of the PA cell. Therefore, the frequency modulation or the chopper frequency should be set at the acoustic resonance frequency of the PA cell, which can be found from the PA resonance curve. To produce the PA resonance curve, we filled the PA cell with one of the standard gases to be detected and set the laser grating to the respective line correspond to the strongest PA signal of the gas. The chopper frequency was then varied, and the PA signal was detected and measured by the microphone. The measured PA signal was normalized concerning laser power.

**Figure 3:**
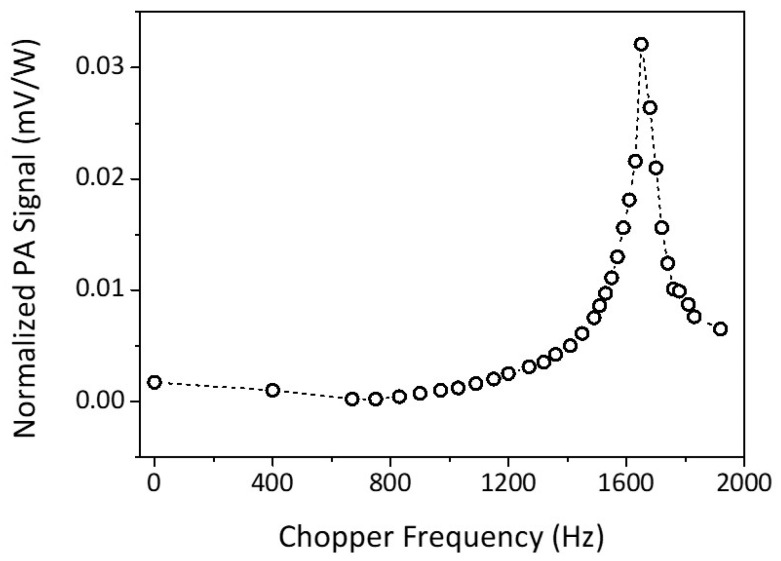
Acetone gas PA signal resonance curve at line 10P20.

Figure 3 shows the resonance curve of the PA cell using acetone as the standard gas, which has a resonance frequency at (1650 ± 5) Hz. The same resonance frequency is also found in the case of ethylene and ammonia. The *Q* value can be obtained from the resonance curve. The greater the *Q* value, the better the photoacoustic cell [36]. The quality factor can be used as a measure of the power loss during the production of the standing waveform from the acoustic waves of every wave cycle [37]. The loss occurs typically as the result of heat conduction and viscosity. In some experiments, loss factor may also result from small leaks on the microphone’s installation joint or other sources, diminishing the value of *Q* factor [37]. From Figure 3, we found the quality factor for the acetone gas at line 10P20 is (27 ± 4). For the other two gases, the ethylene at line 10P14, and the ammonia at line 10R14, we found the *Q* values are (31 ± 6), and (45 ± 10) respectively.

The noise in the instrument comes mainly from the electronics, i.e., from the microphones and the lock-in amplifier. To measure the noise, we ran the instrument with the CO2 laser is off, and no gases in the PA cell. We measured the noise by measuring the signal from the microphone picked up by the lock-in amplifier, with only electronics running. The noise measured at the resonance frequency was 0.31 $\mu V/\sqrt{\mathrm{Hz}}.$ This noise determined the lowest detection limit of gas once we got the linear calibration factor and converted the normalized noise voltage into the gas concentration.

To measure the background signal, the PA cavity was filled with inert gas concerning the CO_2_ laser (i.e., the N_2_). With the CO_2_ laser running, and the chopper set at the resonance frequency of the PA cell, we measured the background acoustic signal detected in the microphone. This background signal came primarily from the laser power absorption in the PA cell windows. We obtained the background signal normalized to the laser power for the line 10P14, 10P20, and 10R14 were 3.79 µV/W, 4.31 µV/W, and 4.52 µV/W, respectively. These background signals were subtracted from any PA-signal measurement in those laser lines.

Our PAS system is capable of doing a multi-component measurement, where there is a mixture of several gases inside the PA cell. In this multi-component setup, for each laser line, the generated PA signal is the linear sum of the PA signal from several trace gas components. Thus, the normalized PA signal (the PA signal divided with the laser power for that line) can be written as [38]

(1) $${\left(V/P\right)}_{i}=\sum_{j=1}^{N}K_{ij}C_{j},$$

where ${\left(V/P\right)}_{i}$ is the normalized PA signal for the i-th laser line, $C_{j}$ is the concentration of the j-th trace gas, and $K_{ij}$ is the linear calibration factor, which is proportional to the PA absorption coefficient of the j-th trace gas at the i-th laser line times the PA cell responsivity.

**Figure 4:**
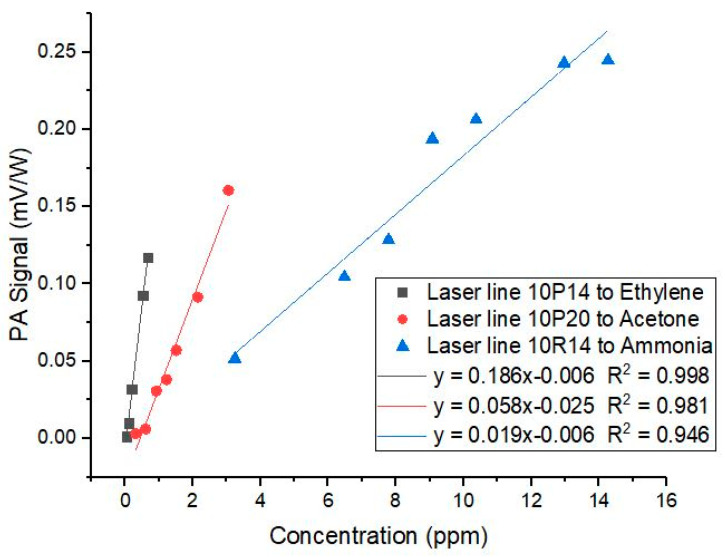
Linearity curves for the normalized PA signal versus ethylene, acetone, and ammonia concentration at 10P14, 10P20, and 10R14 lines, respectively.

To obtain the multi-component matrix calibration, we plotted the linear response of the PA signal concerning the gas concentration. The CO_2_ laser was set at the main absorption line for one of the gas, and the PA signal was then measured for the various concentration of the three gases. The linearity curve of ethylene, acetone, and ammonia gas for the PA signal at 10P14, 10P20, and 10R14, respectively, are given in Figure 4 (there are other linearity curves for each laser line concerning the other two gases that we do not show in the figures). The slopes of the linearity curves were then used to construct the multi-component matrix calibration.

The gradients of the linear relation between the normalized PA signal and the gas concentration were used to obtain the calibration factor *K*_ij_ in Equation 1. We obtained the following relation

(2) $$\left(\begin{array}{c} {\left(S/P\right)}_{1}\\ {\left(S/P\right)}_{2}\\ {\left(S/P\right)}_{3} \end{array}\right)=\left[\begin{array}{ccc} 0.186 & 0.007 & 0.0011\\ 0.011 & 0.058 & 0.004\\ 0.003 & 0.0007 & 0.019 \end{array}\right]\left(\begin{array}{c} C_{1}\\ C_{2}\\ C_{3} \end{array}\right),$$

where (*S/P*)_1_, (*S/P*)_2_, and (*S/P*)_3_ are the normalized PAS signal for the 10P14, 10P20, and 10R14 lines, respectively (in mV/W). While *C_1_*, *C_2_*, and *C_3_* are the concentration of the ethylene, acetone, and ammonia gases, respectively (in ppbv). Inverting the matrix in Equation (2) above, we have the relation for the gas concentration as a function of the measured normalized PA signal, as follows

(3) $$\left(\begin{array}{c} C_{1}\\ C_{2}\\ C_{3} \end{array}\right)=\left[\begin{array}{ccc} 5.416 & -0.614 & -0.171\\ -1.011 & 17.40 & -3.972\\ -0.704 & -0.557 & 52.80 \end{array}\right]\left(\begin{array}{c} {\left(S/P\right)}_{1}\\ {\left(S/P\right)}_{2}\\ {\left(S/P\right)}_{3} \end{array}\right).$$

Equation (3) can be used to determine the concentration of ethylene ($C_{1}$), acetone ($C_{2}$), and ammonia ($C_{3}$) in each breath sample, based on the measured normalized PA signals. The CO_2_ laser is then tuned into line 10R14, 10P14, and 10P20 for the measurement of each sample to get the corresponding PA signal. For each line, the measured PA signal (after amplified by the lock-in amplifier) and the laser power will give us the normalized PA signal (*S/P* = PA signal over laser power). The normalized PA signal is then used to obtain the acetone, ethylene, and ammonia concentration of the breath gas samples. Using the matrix in Equation (3) and the noise level obtained above, we found the lowest detection limit for ethylene is 6 ppbv, for acetone is 11 ppbv, and for ammonia gas is 31 ppbv.

**Figure 5:**
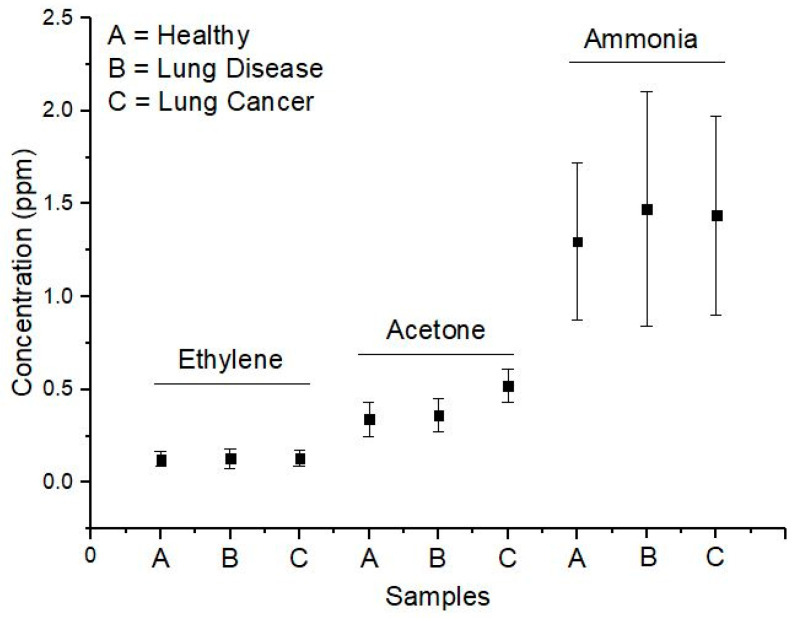
The means and the standard deviations of the ethylene, acetone, and ammonia concentrations for all patients in the three groups.

The means and the standard deviations of the ethylene, acetone, and ammonia concentrations for all subjects in the three groups are presented in the form of graphics in Figure 5. We performed the Student’s t-test for the two means of any two groups for acetone, ethylene, and ammonia concentrations. The results are given in Table 1. From Table 1, we can conclude that there was no significant difference among the three groups for the case of the ethylene and ammonia concentration in their breath. Oppositely, there was a significant difference in the concentration of acetone for patients who have lung cancer, compared to the other two groups.

The use of a CO_2_ laser PAS system for detecting trace acetone in human breath is a novelty that worth to be studied further. Base on the available IR absorption data of acetone, there is no significant absorption line of acetone in the 9–11 µm region of the CO_2_ laser. From our study, however, it turns out that acetone did produce significant PA signals in almost all the CO_2_ laser lines, with the strongest being at 10P20, as shown in Figure 2. Since the strength of the PA signal in Figure 2 seems to be proportional to the CO_2_ laser power, there may be hidden broadband IR absorption lines for the acetone in the 10 µm region. Nevertheless, the fact that we got the linearity curve for the acetone (as in Figure 4) shows the validity of our result: that trace acetone concentration can be measured using a CO_2_ laser PAS system.

Moreover, the validity of the ability of our CO_2_ laser PAS system to measure trace acetone can also be seen from the successful application of our system for measuring the acetone in human breath. Our measurement results for the acetone concentration in the healthy peoples fall in the range 200–450 ppbv. This result is somewhat smaller than the measurement range obtained by Wang and Sahay in [7], in which they reported that the acetone concentration in the breath of the healthy people varies from 0.39 ppmv to 0.85 ppmv. For the acetone concentration in the breath of patients with lung cancer, our result falls in the range 400–720 ppbv, while for the patients with other lung diseases, the range is 222–487 ppbv. These three ranges are still inside the typical acetone concentration range in normal human breath [26]. Thus, although the above statistical test showed that there is a significant difference between the acetone concentration in the lung cancer group and the other two groups, but to claim acetone as a potential biomarker for lung cancer disease still needs further studies. 

For the ethylene and ammonia concentrations, our results for the three groups of subjects do not show any significant difference. The ethylene concentrations from the three groups fall in the range 39–201 ppbv, while the ammonia concentrations from the three groups fall in the range 685–2364 ppbv. The ethylene concentrations range that we obtained is somewhat larger than the typical concentrations in the healthy human. Meanwhile, the ammonia concentrations fall in the same typical range of concentrations in the healthy human.

## 4. Conclusions

Using a high-power CO_2_ laser PAS system, we were able to measure the acetone concentration in the human breath together with measuring the ethylene and ammonia concentrations. Although acetone only has small absorption coefficients in the 10P and 10R CO_2_ laser lines, we found a strong PA signal in almost all lines of CO_2_ laser around the 10 µm region. We applied our system for measuring acetone, ethylene, and ammonia in three groups of people: Lung cancer patients, patients with other lung diseases, and healthy peoples. This multi-component capability is another advantage feature of our PAS system. For those three gases, the CO_2_ laser PAS can measure the three VOCs concentration up to the ppbv level, with the lowest detection limit are 6 ppbv, 11 ppbv, and 31 ppbv for ethylene, acetone, and ammonia, respectively. There is no significant difference in the concentration of ethylene and ammonia among those three groups (with *p*-value > 0.1). While for acetone, we found a significant difference in its concentration between the lung cancer group and the other two groups (with p-value < 0.01), i.e., the patient with lung cancer has a larger concentration of acetone in their breath compared to the other two groups.

## Figures and Tables

**Table 1:** The Student’s t-test result for any two groups for the concentration of acetone, ethylene, and ammonia.

Groups Compared for Each Gas	T-Test	P-Value	Note
**Acetone**			
Lung Cancer vs. Healthy	4.47714	0.000258	Significant at p < 0.01
Lung Cancer vs. Other Lung Disease	3.92928	0.000983	Significant at p < 0.01
Healthy vs. Other Lung Disease	0.53065	0.602526	Not significant at p = 0.05
**Ethylene**			
Lung Cancer vs. Healthy	0.27623	0.785351	Not significant at p = 0.05
Lung Cancer vs. Other Lung Disease	0.03193	0.974876	Not significant at p = 0.05
Healthy vs. Other Lung Disease	0.19831	0.845155	Not significant at p = 0.05
**Ammonia**			
Lung Cancer vs. Healthy	0.66217	0.515817	Not significant at p = 0.05
Lung Cancer vs. Other Lung Disease	0.13711	0.892466	Not significant at p = 0.05
Healthy vs. Other Lung Disease	0.72381	0.479025	Not significant at p = 0.05
